# Rethinking the Difficult Patient: Formative Qualitative Study Using Participatory Theater to Improve Physician-Patient Communication in Rheumatology

**DOI:** 10.2196/40573

**Published:** 2023-03-06

**Authors:** Jerik Leung, Avira Som, Lily McMorrow, Lisa Zickuhr, John Wolbers, Karen Bain, Julia Flood, Elizabeth A Baker

**Affiliations:** 1 Behavioral, Social and Health Education Sciences, Rollins School of Public Health, Emory University Atlanta, GA United States; 2 Department of Medicine Washington University School of Medicine in St. Louis St. Louis, MO United States; 3 Division of Rheumatology Department of Medicine Washington University School of Medicine in St. Louis St. Louis, MO United States; 4 Metro Theater Company St. Louis, MO United States; 5 Behavioral Science and Health Education College for Public Health and Social Justice Saint Louis University St. Louis, MO United States

**Keywords:** physician-patient communication, arts-based education, social determinants of health, rheumatology, concordance, communication, participatory theater, health equity, physician education, interactivity

## Abstract

**Background:**

Effective physician-patient communication is crucial for positive health outcomes for patients with chronic diseases. However, current methods of physician education in communication are often insufficient to help physicians understand how patients’ actions are influenced by the contexts within which they live. An arts-based participatory theater approach can provide the necessary health equity framing to address this deficiency.

**Objective:**

The aim of this study was to develop, pilot, and conduct a formative evaluation of an interactive arts-based communication skills intervention for graduate-level medical trainees grounded in a narrative representative of the experience of patients with systemic lupus erythematosus.

**Methods:**

We hypothesized that the delivery of interactive communication modules through a participatory theater approach would lead to changes in both attitudes and the capacity to act on those attitudes among participants in 4 conceptual categories related to patient communication (understanding social determinants of health, expressing empathy, shared decision-making, and concordance). We developed a participatory, arts-based intervention to pilot this conceptual framework with the intended audience (rheumatology trainees). The intervention was delivered through routine educational conferences at a single institution. We conducted a formative evaluation by collecting qualitative focus group feedback to evaluate the implementation of the modules.

**Results:**

Our formative data suggest that the participatory theater approach and the design of the modules added value to the participants’ learning experience by facilitating interconnection of the 4 communication concepts (eg, participants were able to gain insight into both what physicians and patients were thinking about on the same topic). Participants also provided several suggestions for improving the intervention such as ensuring that the didactic material had more active engagement and considering additional ways to acknowledge real-world constraints (eg, limited time with patients) in implementing communication strategies.

**Conclusions:**

Our findings from this formative evaluation of communication modules suggest that participatory theater is an effective method for framing physician education with a health equity lens, although considerations in the realms of functional demands of health care providers and use of structural competency as a framing concept are needed. The integration of social and structural contexts into the delivery of this communication skills intervention may be important for the uptake of these skills by intervention participants. Participatory theater provided an opportunity for dynamic interactivity among participants and facilitated greater engagement with the communication module content.

## Introduction

### Background

Patient-physician interactions represent a pathway through which health differences manifest [1]. Interactions that make patients feel rushed, disrespected, or unheard reduce patient satisfaction and health outcomes [2], whereas those that demonstrate respect for patients increase their adherence to prescribed medication regimens, lead to more consistent follow-up, and improve their health outcomes [3]. Marginalized people (eg, those who identify with nondominant social identities such as race, gender, sexual orientation, gender expression, or class) are more likely to experience poor interactions [4].

Communication facilitates effective physician-patient interactions, enhances patient empowerment, improves patient understanding of health conditions, and enriches the therapeutic alliance between patients and practitioners. Effective communication has been linked to positive physical and mental health outcomes [2,5], thus making it a component of health care for all patients and is especially critical among those with chronic diseases because of the frequency of interaction between physicians and patients [6].

Patients with the chronic condition systemic lupus erythematosus (SLE) report challenges when communicating with their health care providers [7]. SLE is an autoimmune disease that follows an unpredictable course and disproportionately affects women of color [8]. Nonspecific symptoms and unpredictability of the syndrome create communication barriers with physicians [9]. In addition, medical mistrust among marginalized patients further strains physician-patient communication [10], because the health care professionals may not know how to best address it or how to recognize the social determinants of health (SDOH), which can contribute to what some have deemed contextual medical error (error in medical decision-making derived from overlooking patient context) [11]. Similarly, bias based on socioeconomic status exhibited by providers or other members of the health care system can also present challenges for patient-provider communication [12] by, for instance, relying on culturally produced stereotypes of individuals with low socioeconomic status [13,14]. These factors strain the therapeutic relationships between individuals with SLE and providers, thereby contributing to disparities in SLE outcomes [15].

Concordance reflects the degree to which patients and practitioners agree with the treatment plan [16,17]. A core component of concordance is empathy [18], which can be divided into 3 domains: cognitive, emotional, and behavioral. Although the cognitive and emotional domains are focused on understanding another person’s perspective (cognitive) and internalizing those feelings (emotional), behavioral empathy is the ability to express the *(internally) experienced (cognitive and emotive) process* [19]. The acquisition of these different components of empathy requires different teaching modes. For instance, instructional sessions focused on cognitive and emotional empathy may use patient narratives to teach recipients how to understand others’ situations or perspectives regarding living with a chronic disease. To improve behavioral empathy, educators might use patients who have been standardized to build physicians’ verbal and nonverbal communication skills to convey their understanding of patient experiences and viewpoints.

Interventions that address patient communication among physicians need to be tailored to the needs of specific vulnerable populations, both in the nature of disease and in the multitude of contextual factors that shape people’s abilities to engage with their health care providers [2]. Skills training for physicians often relies on information-sharing modules accompanied with observed practices with actors or patients [20]. The format of these sessions may hamper the incorporation of the patient context, the structural and social factors that influence that context, or how to address them during clinical encounters. To explore these topics, participants need a safe learning environment to ask questions about potentially sensitive topics, as well as opportunities to ask questions about the impact of their approach with the actual or standardized patient. Arts-based approaches to education are particularly well positioned to meet these needs because they facilitate active engagement among participants [21], particularly on social issues [22]. Given the relative novelty of arts-based interventions for physicians and medical trainees, a formative evaluation approach incorporating participant reflections is important for intervention refinement.

### Objective

The purpose of this study was to develop, pilot, and conduct a formative evaluation of an interactive arts-based communication skills intervention for graduate-level medical trainees grounded in a narrative representative of the experience of patients with SLE. The results reported in this paper discuss the immediate impact of the intervention on participant perceptions of this mode of communication skills training and ways to improve the curriculum for future implementation.

## Methods

### Participants

We used a convenience sampling strategy for recruitment. All trainees in the rheumatology fellowship program at an academic medical center attended the session as a part of their required conferences (n=7). Internal medicine residents rotating through the specialty of rheumatology were also invited to attend (n=1). All studies were conducted in accordance with the relevant guidelines and regulations. Verbal informed consent was obtained from all the participants.

### Conceptual Model

**Figure 1:**
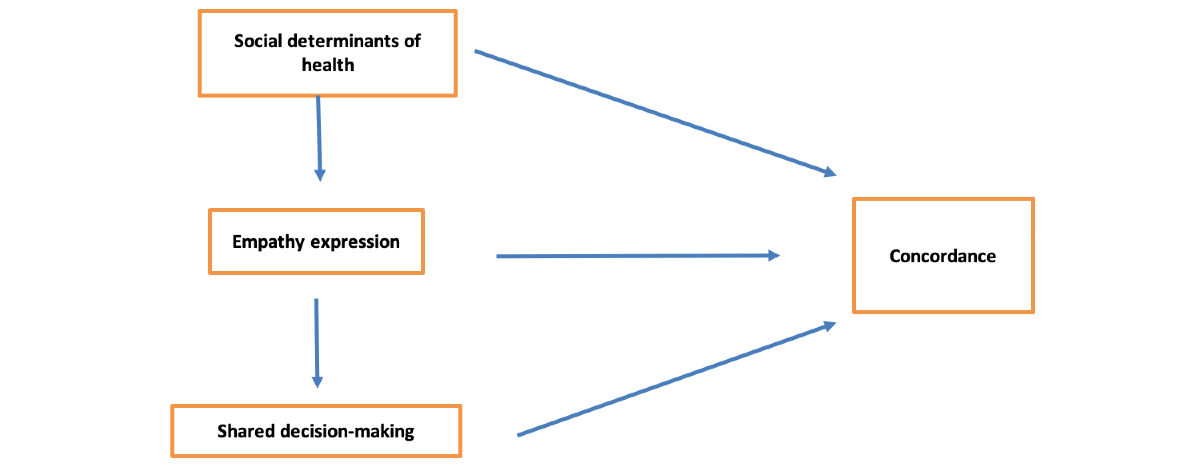
Conceptual model. We posited that each of these concepts are independently associated with concordance and also build on each other. Understanding of social determinants is seen as cognitive empathy, and the ability to move that toward behavioral empathy requires additional skill building. The increased understanding, and ability to express it, is then seen as leading to the ability to better engage with patients in shared decision-making processes, which in turn contributes to provider-patient concordance.

The intended outcome of the session was to alter physicians’ attitudes toward concordance and provide introductory skills training that might lead to better communication and concordance with patients during routine interactions. Through discussions with patients and health care providers and a review of the communication skills training literature, we identified 3 key concepts: SDOH, expression of empathy (behavioral empathy), and shared decision-making (SDM; Figure 1). Importantly, the SDOH module consisted of how providers may engage with various SDOH with their patients and group discussions on how to best facilitate connection to resources for patients when necessary.

### Intervention Development

We conducted a literature review to create interactive modules that align with the 3 components of the conceptual model (SDOH, empathy expression, and SDM) [23-27]. For delivery of the intervention content, we worked in partnership with the Metro Theater Company, a nonprofit educational theater organization. The research team collaborated with the members of the organization to develop a script for the initial scenario that depicted a physician-patient conflict and reflected a typical experience of living with SLE (eg, patients who were feeling that they were not being heard and physician were ignoring patient concerns). The actors playing the physician and patient had expertise in improvisation, which allowed for dynamic interaction with audience members. In addition, the research team assisted in preparing the actors for SLE-specific issues by providing relevant background information and feedback from both physicians and patients during rehearsals.

### Ethics Approval

This study was approved by the Washington University Institutional Review Board (protocol #202010105).

### Intervention Description

The intervention consisted of a 120-minute session with a mixture of didactic and interactive modules. Although originally designed for in-person learning, the intervention was adapted to web-based learning and delivered through videoconferencing software because of the COVID-19 restrictions. The videoconferencing environment functioned as the main “forum” of the forum theater. The primary adaptation made in the videoconferencing environment was that one member of the study team (JL) used the *spotlight* feature throughout the session so that only those speaking were visible to all (eg, actors during the actual scenes). All the participants were visible during the hot seating portion of the initial scenario. There were 5 phases of intervention, as described in Textbox 1.

**Intervention Details**

**Overview**

- Phase 1 used a forum theater approach and presented a scenario to participants via a prerecorded video depicting actors playing out a consultation between a physician and patient with lupus. After the viewing of the video, the participants had the opportunity to ask the physician and patient actors questions. Phases 2-4 engaged participants in interactive modules, each focusing on a theme from the conceptual model (eg, social determinants of health, empathy expression, and shared decision-making). Phase 5 consisted of a return to the initial scenario and opportunity for participants to generate ways to replay the scenario and actively engage with in-character actors to test out strategies.

**Phase 1: Initial scenario presentation and “Hot Seating”**

- Phase 1 presented the initial scenario, which served as a central grounding point for the entire intervention. The scenario was designed around common issues of lupus with 3 key cut points, which aligned with the 3 themes (social determinants, empathy, and shared decision-making) in the interactive modules (phases 2-4). The initial scenario was video recorded and presented to participants. After presentation of the video, the characters in the video joined the Zoom call and participants were lead through a facilitated dialogue during which they could ask characters about rationale, why they made certain decisions, and why the scenario happened the way it did. A physician member of the research team (LZ) was also available to “feed” information to physician actor during the intervention, in case participants asked medical questions beyond the scope of the background provided during rehearsal. This ensured the believability of actor performance.

**Phase 2: Social determinants of health board game**

- Phase 2 presented an adapted version of the social determinants of health board game. Participants were divided among 4 characters, each with a lupus-specific background (ie, duration of disease, relative severity, and organ complications) and varying degrees of advantage owing to a number of social determinants of health (ie, socioeconomic status, gender, sexual orientation, and race). These characteristics determined the number of initial “vitality chips” for each character. Each team took turns in rolling a game die that corresponded to advancement through the pathway on the game board. After each advancement, players encountered a card that either added or subtracted vitality chips based upon the initial background of lupus symptoms and social factors.

**Phase 3: Empathy expression role-playing activity**

- Phase 3 presented didactic material on empathy and several tools to facilitate behavioral empathy skill development. Participants were divided into pairs, with each group focusing on a different behavioral empathy tool. Multiple tools were provided to account for different learning styles and varying scenarios with patients. Each group was facilitated by a member of the research team. Participants read through the tool description components, discussed in subgroups, and then role-played with each other, assisted by facilitator.

**Phase 4: Shared decision-making**

- Phase 4 presented didactic material related to shared decision-making. Participants read through shared decision-making steps and key phrasing suggestions. As a group, participants then clarified any question and were asked to summarize the different steps presented by the didactic material.

**Phase 5: Strategies**

- In phase 5, participants were guided through a facilitated activity with the Metro Theater Company. Participants rewatched original scenario but this time breaking at key cut points. Participants were then asked to generate alternative strategies for engaging, drawing from the 3 modules in which they had just participated. Some participants were then asked to role-play with the patient actor to try out the identified strategies.

### Pedagogical Approach

We used an arts-based participatory theater approach to deliver the intervention. Participatory theater is grounded in the empowerment theory by Paulo Freire [28] and the initial Theater of the Oppressed by Augusto Boal [29]. This leverages the knowledge of disenfranchised people to transform oppressive structures by inviting members from the audience to participate in the performance and by asking them to envision ways to transform their current social realities. Audiences first engaged with performers and facilitators to represent the reality of their current experiences and then cocreate ways to modify these conditions using theater techniques as a vision to achieve change [29,30]. Actors play various parts outlined in the script in the actual application of this participatory theater approach. A facilitator engages the audience and allows for stoppage and replaying of scenes and prompts members from the audience to provide suggestions on how to redo interactions for different outcomes. Audience members are then asked to tap into the scene and take on the role of the characters (replacing the actors). This differs from standardized patients in medical education in that the trainees typically only play the role of health care provider and do not necessarily have in-built facilitation or structured observation [31].

### Measurements

Participants shared feedback through 2 focus groups facilitated by members of the research team during the last 20 minutes of the session. Facilitators asked participants about the strengths and weaknesses of the intervention’s design and implementation as well as suggestions for improvements. Focus group questions were provided to the participants by the authors.

### Analysis

We conducted a qualitative analysis using a constructivist paradigm. The entire session was audio recorded. Focus groups and discussions for each phase were transcribed verbatim and analyzed using focused coding techniques [32], in which multiple members of the research team independently assigned codes to transcripts based on the interview topics, discussed disagreements, and arrived at a consensus on the best way to address discrepancies. After the initial coding, codes were arranged into clusters with summary paragraphs describing key elements of each cluster, along with supporting quotations from the focus groups. The focus groups were audio recorded and transcribed. To maintain confidentiality, audio files and corresponding transcripts were stored on a password-protected computer. Complete anonymity could not be maintained with the audio recordings because voices could potentially be identified; however, the participants did not state their names during the focus groups. In addition, unique identifiers were generated to label the speakers in the transcript documents. Participants were not compensated for their participation.

## Results

### Overview

A total of 8 trainees participated in the workshop (Table 1). All the 8 participants also participated in the postsession focus groups (4 participants per group) and the demographic questionnaire. The participants’ ages ranged from 26 to 40 years, with the majority (6/8, 75%) being aged between 31 and 35 years, women (5/8, 63%), and non-Hispanic or Latinx (7/8, 87%).

**Table 1:** Participant characteristics.

Demographic variables			Value, n (%)
**Age group (years)**			
	26-30	1 (13)	
	31-35	6 (75)	
	36-40	1 (13)	
**Gender**			
	Woman	5 (63)	
	Man	3 (37)	
**Hispanic or Latinx**			
	Yes	1 (13)	
	No	7 (87)	
**Race**			
	White	2 (25)	
	Asian	3 (38)	
	Other	3 (38)	
**Medical training**			
	First year fellow	4 (50)	
	Second- or third-year fellow	3 (38)	
	Resident	1 (12)	

### Focus Groups

#### Overview

We grouped participant feedback into 3 categories as follows: value added by method of content delivery, suggestions for improvements in the method of delivery, and overall reflections.

#### Value Added Through Method of Content Delivery

Participants generally thought that the opportunity to deeply investigate the interaction between the patient and provider, rather than just observing it, added value to their learning experiences. They found value in being able to ask questions of both the patient and provider after the videotaped interaction, something they had not been able to do either in prior education settings or in actual interactions with patients (Table 2; quotation [Q] 1).

The ability to ask questions to both the patient and provider and have each respond while maintaining their role functioned as a type of confessional interview that allowed the respondents to hear the internal narrative of both the patient and provider. This was an important experience for providers who often walked out of a room and asked themselves what the patient took from the interaction but did not have the opportunity to ask the patient (Table 2; Q2).

**Table 2:** Formative evaluation feedback.

Categories	Quotations
Value added through method of content delivery	1. Yeah. I would say I got a lot of value out of being able to ask questions, uh, after that first stage, that initial interaction. I think that was, you know, something that I don’t really ever get to do. Sometimes we get that information [from patients] through a survey results and things like that, but it’s really interesting. 2. ...I thought it would be...nice that—kind of get the answers from those two [physician and patient actors] just for that perspective, because sometimes I think it—or I guess it offered a time to ask questions that maybe sometimes I walk out of the room and I’m like, “Huh, I wonder, uh, did they get that, or did I totally miss it?”...you know, you just keep moving because you have to, but I thought that that was a unique experience... 3. I thought that was great, actually, that they [actors] were willing to give their time to, you know, shoot that video in advance and then, after the fact, let us play role and do those breakout sessions. I felt like that was more of a believable, you know, standardized patient interaction... 4. ...I tend to like how—observing how other people interact with patients because I think in training very often we’re just sort of “go do it” and you never actually get to see someone role modeling those behaviors... 5. Yeah, so with medical school, we had standardized patients. And then residency is the same thing when we were running mock codes or what not...and so they [instructors] would have this little blurb of who they [standardized patients] would be, and you would walk in to the room and just play role that. But there’s not really someone who would show you how that interaction, like may have occurred—right, or should have occurred. It is just “This is what you’re supposed to do,” and then they would correct you after the—or give you feedback after the fact... 6. [Question for physician-actor]...But do you think the interaction would have been different if she [patient-actor] had shown up on time? Like, maybe it would’ve helped to have had a shared agenda at the beginning? 7. (Participant speaking)...when she [patient-actor] came 25 minutes late, you would have 5 minutes before your next patient...So sometimes I have taken the tact of, like, okay, like, I’m still—you made the effort to come here. I’m making the effort to see you, but I can’t, you know, make my other patients have a negative outcome because you were late. So if I had asked you to, maybe, like—I’m gonna try to fit you in as best as I can, but you may have to wait, like, until I have a free slot in my schedule. How would you [patient-actor] have reacted?... 8. (Patient-actor speaking): I’m walkin’ out. And that-that sucks because, like you’re [participant] saying, you’re doing the best that you can to stay on schedule ‘cause you do have other patients. And the truth is, just because I’m late, you can’t let that ripple out to everyone else. You can only be so late to everyone else...but I’m still waking out. Um, but I think that [a physician] putting the [emphasis on], uh, “But I really do want to see you. So if you can stay, we’ll try to get you in today. Otherwise, let’s really look at what day you have avail-available, and let’s plan ahead.” So, then, it’s like, okay, [the physician might say] let’s look at another two weeks ‘cause I really want you to have enough time to plan to get here and really wanna give you, um, the time that you need so that I can, like, focus on you. Um, ‘cause showing that, you really are important, and I really do wanna see you. And...I can glance at your rash now, but I really want you to do a follow-up so that I can go even deeper... 9. I thought it was useful for demonstrating, you know, the impact and how—sort of the trends of...acquiring more and more social determinants of health in a positive or negative direction. 10. ...I think it was a good exercise, especially for, like, visual people. It was, like, really nice to see, like, those little chips and see them taken away. I think it makes you understand that one person can have two different outcomes that are very polar depending on things that we don’t necessarily—maybe may not think about first, like, you know, one—you know, the same person having social support versus no social support. Outcomes are very different... 11. ...just to be able to use a couple of different tools [was helpful], ‘cause I think each tool may or may not work for the particular person who’s using them. 12. I think the verbiage and examples that were used in that chart are excellent, and I—absolutely see myself using them, uh, and practicing them because I admittedly am not smooth in working through those three phases and, uh, I think...that’ll be really helpful for me, so... 13. Um, and I think one thing I’ve taken away from today is, like, the Ask-Tell-Ask session—is, um, to Ask-Tell-Ask every concern as it comes up and not wait ‘til the end—to kind of hit them [with] all this information that they may not be able to absorb all at once... 14. I think it was a good exercise...getting to kind of...walk back into the bad encounter, kind of apply the strategies we’ve learned...I mean, we practiced the NURSE strategy in the breakout room, and I think that was really helpful. I mean, we do a lot of those things [exercises] separately. We put it in—insert it into our conversations [with patients] somewhere but never really have a systematic approach to it. So I think learning about that, too, and practicing was helpful.
Suggestions for improvements in method of delivery	15. ...I think continuing the trend of being interactive and things like the rest of the session...somehow would have been nice too 16. Um, I think where I got lost for a little bit is when I read—I tend to do better when I both read and hear or—sort of—the reading I often just glaze through really quickly [laughter], and then how much it-that it sticks or not you just have to find out later [laughter]. So I think for me the reading portion...if there was some other sort of cue like he read it as we read it, or just reading certain portions...I just know I personally don’t do as well with the just read it on your own kind of thing. 17. ...I thought it would have been nice if we sort of had a little bit more discussion after that segment [empathy expression], maybe like what aspects of each tool, like, a particular group is using that they found useful or not so useful, um, especially if you don’t have time to use all three. Then you get to hear a little bit of some other tools. 18. Um, I think for me it was sort of—I-I felt like our group maybe didn’t, uh, wasn’t as interactive for that part [SDOH game], and maybe it is for some of the reasons that [participant] had mentioned. But also, um, I think when we were reading the card it was just a lot of words and information, um, and so, like, trying to respond to too much may have inhibited a little bit of the—or at least not being able to see the card on the screen. That might have made a difference too. Um—in-instead of just being read, so that-that may be another option to sort of, uh, get more interaction. 19. I thought it [role-playing] was helpful. One thing that I think was a little challenging for me was I wasn’t really sure what to say as the patient, when I was role playing as the patient, just ‘cause some of the initial video, kind of outlined all of [the patient’s] thoughts and her actual symptoms, so I didn’t really remember all the details from it [while role-playing]. 20. ...when we did this as residents, we had, like, little actor notes that would tell us how exactly obnoxious we should be or, you know, like, what we shouldn’t do, what we should say...just like little cues...
Overall takeaways—positive	21. So given everything else that we were tryin’ to accomplish during this whole session I would have—I-I don’t know that I found that—that it added a ton of knowledge for me. But in different groups I think it could have—be important... 22. So, during residency, we kinda had things like this too. But I think it’s a little bit different looking at it from the [rheumatology] point of view, as opposed to internal medicine as a whole, ‘cause I feel like, with rheumatology—and I guess with every disease but, I feel like, more so with things...like rheumatologic diseases, the social determinants of health are, I think, even more important. I think...rheumatologists...are, like, the full caregiver for, like, most of our patients, right? If they have a fever, they tell us first before they tell their primary care doctor. So, I think, knowing the social factor that play into their healthcare is more so very important for us to kinda be aware of. 23. I like how the workshop gave us some additional insights into both what the patient and the physician is thinking...because I think a lot of the time we maybe try to figure out how a patient might respond, and there’s no real feedback. We just have to guess and go with something... 24. I think all these tools...go into treating the patients as people first who have medical problems and not medical problems that are associated to a person. So I think it’s important always to have the person be the center of the interaction that hap—a person who happens to have medical issues. And I think, if that’s the focus, you’ll end up catching more social issues that the patients bring to you, and it will help you provide better care. 25. Sure, um, I think for me is, as we see patient in the clinic, especially a lot of time with a busy schedule, sometimes—or by the language can—we sort of focus more on the computer screens instead of looking at the patient and actually sit down, just ask some simple questions, like, “How are you doing?” ...we kind a tend to ignore, uh, the social factors. We focus more on the medicine part of it, uh, but we...we don’t really explore like a—find of a cause or the reason why they [patients] are not taking medication... 26. ...you know, we treat the medical issues, and that’s what we’re there for. But, on the other side, to help with the patient as a whole—their social conditions and...how that affects them, I tend to put on the back burner. But this, you know, this session’s nice to help me bring that back and go, “Okay, well, the—what are we going to be doing, um, in terms of their condition flaring? Why is it flaring? Is it because they don’t have the means for it, and what can we do to help with that?”...just being able to say, “Okay, why—what can we do outside of their medical condition that can help it, um, improve that [flaring]?” 27. ...I think being able to sort of get on the same page from the get go just by simply, you know, introducing and taking a second to do that and make sure you both know each other to start the conversation, that was a big difference. And I think it sort of carried the rest of the conversation. But from there, [participant] did a good job of, like, making her [patient-actor]...[feel heard] regarding her concerns around the rash but, also, you know, some of the other things that are going on as far as just getting to her appointment and some of her life stressors. 28. ...I really think that the most important part of this is to make the patient feel that really listen to their concerns, even if it’s not our [physician]—most important, um, part of that discussion that day... 29. ...[participant] did a good job of just...providing attention to her concern. Um, but, also, you know, in—you know, taking a look at it and kinda going through those steps. But, when she delivered information that maybe the patient wasn’t totally agreeable to, she provided kind of like a back-up to say, “Hey, you know, I think we should try this. But, you know, just so you know, if it doesn’t work, like, let’s keep working towards a solution.” 30. [Participant in role-player] didn’t really provide solutions for those [social stressors], but she [patient-actor], at least, felt like, “Okay, [participant role-player] knows that I’ve been having a day or a week,” or whatever. And they can kind of, uh, move on from the conversation. One of the other big things I think I appreciated that [participant role-player] did was emphasize, um, why [participant role-player] felt the-the medication was important and, um—so she [patient-actor] kinda knew why, like, why does he really care. Like, why don’t we just switch medications? 31. I think that is an important thing to emphasize. Like, let’s really make sure that this rash is from the medication or not because I, you know, I think the medication is important. And that helps her feel like, okay, [participant role-player] not just forcing me on this medication against my will too. So I think that was an additional thing that helped her understand where [participant role-player] was coming from after he had already heard her concerns.
Overall takeaways—concerns	32. Um, I think it can play out as [participant role-player] did with [patient-actor] today. But, um, the other chance it could be, okay, like...how much can this derail the actual shared agenda that you want to get completed during the visit? Sometimes, once—at least, I’ll just say for myself. You know, you have, like, that self-catastrophizing type of mentality. And so, like, one bad thing comes up, and then you’ll just continue to the next bad thing. 33. And sometimes it can just span the whole visit where you’re like, “Well, there was the 30 minutes of just complaints, and I couldn’t get a word in with the patient...” 34. I think they went through everything, and I think [participant] had pointed out something important at the beginning. I don’t think most of the time at least when, uh, options are presented to the patient we might not go through the three steps in one visit. We might have to follow up with the patient on-over the phone or do something else. 35. It’s hard to be thorough with each one of those steps [shared decision-making tool] and still make it under 30 minutes and have examined the patient and do everything.

In comparing the forum theater approach to their previous standardized patient experiences, participants indicated that the forum theater was better because they were able to test alternative strategies for changing the interaction and see how a patient might respond. For instance, the format of letting participants play either the provider or patient role while an actor played the other role was seen as more believable than previous experiences with standardized patients (Table 2; Q3). Participants noted that in prior experiences, they would practice certain scenarios but did not get to see someone role modeling the behaviors (Table 2; Q4). They would then be corrected by the instructor, but the standardized patient typically did not provide feedback or insight into their experience of the provider’s behavior (Table 2; Q5).

The desire to test strategies was further evidenced by the types of questions asked by the physician and patient actors. Questions for the physician actor centered on wanting to know more about why the physician did not ask about the patient being late and whether they thought the interaction would have gone differently had the patient been on time (Table 2; Q6). Participants went further by asking the patients how they would feel if they were asked to reschedule when they arrived at their appointment late (Table 2; Q7). The patient-actor response suggested that they would likely leave and be unlikely to reschedule unless the provider showed that they had taken the patient seriously (Table 2; Q8).

Participants appreciated when visual cues were built into the activities because they helped reinforce the primary message of that activity. For instance, the SDOH board game contains red chips, which are meant to represent an acquisition (or lack of) advantage in society. Participants commented that these chips were helpful in demonstrating the trends and accumulation of both health-promoting and deleterious impacts of SDOH (Table 2; Q9). The red chips provided an opportunity for critical reflection on the forces that led to the accumulation of certain resources that affected patients’ ability to manage their disease (Table 2; Q10).

An additional element of activities that participants found helpful in the training was the presentation of a variety of different tools for the same concept, as well as specific phrasing or verbiage suggestions. The use of a variety of tools enabled participants to begin making connections on how these tools could be used in their practice and future interactions with patients. (Table 2; Q11-12). Participants began to see that there is not just one way to address the issues, but a range of options they can choose from (Table 2; Q13).

Participants praised the interactivity of some parts of the session, particularly the opportunity to interact apply some of the strategies they had practiced throughout the session with the actors (Table 2; Q14).

#### Suggestion for Improvements in Method of Delivery

Although participants were enthusiastic about the training, they also noted that some parts of the session were not quite engaging. For instance, they mentioned that the presentation of some of the didactic components could have used more active ways to engage with written material (Table 2; Q15-17).

Participants also suggested ensuring multiple ways of engaging with information for different activities (ie, both visual and auditory elements). Having multiple methods of engagement would help the participants contribute to a deeper level of interaction during the discussion sections of the session (Table 2; Q18).

Suggestions were also made to make some of the activities more fluid. For instance, during role-playing exercises, participants were not sure what to say when role-playing as a patient, which could make the role-playing feel forced, especially because participants were not used to acting. Without cues or guidance, it was sometimes difficult for participants to tell whether they were fulfilling the goals of the session (Table 2; Q19). One suggestion was to provide actor notes that would guide how participants should try to act (Table 2; Q20).

#### Overall Reflections

In their reflections on the session, participants commented on how the 3 different pieces of the session (SDOH, expressing empathy, and SDM) complemented each other in ways that they had not previously considered (Table 2; Q21).

Participants noted that they were familiar with some workshop concepts. However, it was evident from their reflections that this session went further by building on the foundation that participants may have already had and provided additional context for these terms, such as SDOH, and how having this lens impacts their empathy with patients in the SDM process. The participants noted that this was particularly important given the impact SDOH has on the ability of patients with rheumatological diseases to manage their diseases (Table 2; Q22).

Specifically, participants saw the tools and activities as helpful by providing insight into what the patient and physician were thinking about the same topic. The activities appeared helpful in contextualizing physician-patient interaction and ensuring that physicians see patients as people who have medical problems and not just medical problems associated with the person. For example, physicians tend to focus on getting patients to take their prescribed medications without exploring *why* patients are not taking their medication or adhering to care recommendations (Table 2; Q23-25).

This reframing then encouraged participants to also think about their role as health care providers in taking action to address some of the factors outside of the *medical condition*, which may affect lupus (Table 2; Q26).

This synthesis was further evident in what the participants took away from the session. For instance, they considered framing the beginning of the interaction differently by spending more time with introductions and establishing a rapport with the patient. This underscores that one of the most important parts of the interaction is ensuring that the patient feels that their physician is listening to their concerns and connecting actions directly to those concerns (Table 2; Q27-28).

Participants also connected this rapport building to later points in decision-making with patients. Demonstrating that the physician provided early attention to patient concerns was helpful when delivering information that the patient was not completely agreeable to. Participants noted the importance of having a backup option for a treatment decision and that they would continue working toward an agreeable solution if the initial treatment recommendation was not working for the patient (Table 2; Q29).

Participants similarly saw rapport building as generally leading to more productive discussions in the context of patient life stressors and other SDOH. Although the participants noted that direct solutions to other life stressors cannot always be provided within the context of a clinical visit, attention to these life stressors by physicians is still meaningful if the patient feels heard and strategies are discussed to help the patient and provider get on the same page early (Table 2; Q30). Participants directly connected this early rapport building and acknowledgment of SDOH to being able to better emphasize important elements of lupus management such as the importance of consistently taking medications (Table 2; Q31).

Participants noted some obstacles to implementing the tools presented during the session given the reality of working in a clinical setting. For instance, some participants voiced concerns that asking how patients are doing may open the conversation to issues beyond what they can address. There was a concern that some of these strategies would invite patients to focus on self-catastrophizing behaviors or that some of the strategies would take more time than allotted during a clinical encounter (Table 2; Q32 and 33).

Others have noted that suggestions for interacting with patients might not be realistically implemented in a single patient encounter. Often, a provider may not be able to go through all steps in decision-making in one visit, so it can be challenging to cover each of the SDM processes and examine the patient in a time-limited clinical visit (Table 2; Q34 and 35).

## Discussion

### Principal Findings

We presented formative evaluation data from an interactive, arts-based communication skills workshop for rheumatology trainees. Our qualitative data suggest that participants responded positively to the level of interactivity of some session components, the overall cohesiveness of the separate phases, and the unifying approach of theater-based performance. The design of the session enabled participants to integrate the different components of the sessions, relative to the previous types of workshop training (often compared with standardized patients).

The novelty of this session was the integration of structural and social factors as a context for patient encounters. This was achieved through the intentional sequence of theory-grounded activities built upon each other (Figure 1) and facilitation, which guided participants in linking activities together. This approach allowed participants to move from simply receiving information about communication behaviors to applying that knowledge in a dynamic environment that incorporated patient lived experience. This was evidenced by participants in their generation of strategies: participants discussed the importance of asking how patients were doing at the beginning of the encounter (SDOH activity or empathy expression), which they saw as helping with rapport building and ultimately facilitating the space for cooperative SDM to occur. Although discussions on structural factors and SDOH have permeated medical education and are increasingly large parts of the curricula [33], the integration of these SDOH in communication-specific modules is less common [2]. Our findings suggest that this integration is critical in the uptake of information and skills in both SDOH and communication domains.

The incorporation of the structural context of health inequities was greatly facilitated by the participatory theater approach. The unifying scenario, presented at the beginning and developed in conjunction with an educational theater group, was an important grounding for participants, as they interacted with actors throughout the session. This unifying scenario was received positively by participants, who affirmed that the application of arts-based pedagogy was suitable for graduate medical education. Our findings align with the application of forum theater in other medical settings including general medical education [22] and specific discussions around social factors such as race [34]. In line with these studies, we observed the advantage of forum theater in the exploration and generation of ideas among participants rather than prescribing a particular solution. This was evident in our findings, as participants praised the dynamic interactivity of the theater components, which appeared to be more engaging than their previous experiences in medical education settings with standardized patients.

### Limitations

This study has several limitations. First, we performed only a single session with 8 rheumatology trainees. Although we would have ideally conducted additional sessions, our intention was to pilot materials that were specifically designed for a rheumatology setting to assess what activities did or did not work well. Moreover, within this narrow population, we were able to include all rheumatology trainees at the study institution. An additional limitation is that we relied on a single script in our study. This may limit the scope of information received by participants; therefore, future iterations of this type of intervention may consider developing multiple scripts for the same topical area. Future iterations of this session may also consider designing multiple sessions to track changes in attitudes and integrate evaluation metrics with participant observations in actual clinical settings. In addition, the intervention was conducted via videoconferencing because of the COVID-19 pandemic restrictions, but the intervention may have been received differently had it been in person. Finally, focus group methods were limited (eg, not able to engage in member checking) because of funding, but future versions of this work may consider additional recruitment strategies for more rheumatology trainees (ie, multisite intervention) to obtain a larger sample to better gauge data saturation.

### Conclusions

Our findings from this pilot test of communication modules suggest that participatory theater is an effective method for framing physician education through a health equity lens. Feedback through qualitative focus groups revealed several considerations for the further exploration of this approach and framing. First, a critical insight from participant feedback was the need to acknowledge the workload of the physicians. Participants raised concerns about the reality of working in fast-paced clinical environments and the difficulty of implementing some of the tools. This aligns with the current understanding of the increasing physician loads, more time spent on administrative tasks or electronic medical record [35,36], and pressure to see high volumes of patients while also providing optimal care management [37]. The prospect of adding additional tasks to an encounter, such as spending time discussing social factors with patients, is daunting. This suggests that education sessions such as the one presented here need to balance the realities of clinical practice with the new knowledge from these sessions. These insights also suggest that complementary sessions designed to educate patients about the demands of physicians are necessary, using similar tools and approaches as those described here. This suggestion is in line with work that has pointed to the disproportionate emphasis of interventions on physician behavior, but which misses an important half of the physician-patient dyad [38].

In addition, our intervention incorporates elements of “structurally competency,” which Metzl and Hansen [39] define as the “ability to discern how a host of issues defined clinically as symptoms, attitudes, or diseases also represent downstream implications of a number of upstream decisions about such matters as health care and food delivery systems, zoning laws...” Structurally competent approaches to medical education prioritize training medical professionals to recognize the ways in which structural (upstream) factors shape the medical encounter and patient outcomes (downstream). The evidence of the basis of this work, supported by our formative findings here, suggests that these structurally competent approaches are effective at integrating SDOH education. Moreover, it suggests that this approach can also facilitate concordance between physicians and patients, a bedrock for a productive therapeutic alliance, and improvement of health outcomes.

## Abbreviations

SDM: shared decision-making
SDOH: social determinants of health
SLE: systemic lupus erythematosus
Q: quotation

## Data Availability

Qualitative data analyzed are not publicly available because of the limited number of participants in this pilot study and the possibility of reidentification from raw, anonymized transcripts. These data can be provided by the corresponding author upon reasonable request.
